# Materials degradation in non-thermal plasma generators by corona discharge

**DOI:** 10.1038/s41598-021-03447-w

**Published:** 2021-12-17

**Authors:** Mar Cogollo de Cádiz, Adrián López Arrabal, Andrés Díaz Lantada, M. V. Aguirre

**Affiliations:** 1 Product Development Laboratory, Department of Mechanical Engineering, Escuela Técnica Superior de Ingenieros Industriales, Universidad Politécnica de Madrid, C/José Gutiérrez Abascal, 2, 28006 Madrid, Spain; 2 Research & Development Department, Cedrión, Consultoría Técnica e Ingeniería S.L, 28919 Leganés, Spain; 3 Department of Aerospace Materials and Production, Escuela Técnica Superior de Ingeniería Aeronáutica y del Espacio, Universidad Politécnica de Madrid, 28040 Madrid, Spain; 4 Centro de Investigación de Materiales Estructurales (CIME), Madrid, Spain

**Keywords:** Electrical and electronic engineering, Plasma-based accelerators, Scanning electron microscopy, Corrosion, Metals and alloys, Electronic properties and materials

## Abstract

Atmospheric corona discharge devices are being studied as innovative systems for cooling, sterilization, and propulsion, in several industrial fields, from robotics to medical devices, from drones to space applications. However, their industrial scale implementation still requires additional understanding of several complex phenomena, such as corrosion, degradation, and fatigue behaviour, which may affect final system performance. This study focuses on the corrosive behaviour of wires that perform as a high-voltage electrode subject to DC positive corona discharge in atmospheric air. The experiments demonstrate that the non-thermal plasma process promotes the growth of the oxidative films and modifies the physicochemical properties of the materials chosen as corona electrodes, hence affecting device operation. Surfaces exposed to this non-thermal plasma are electrically characterized by negative exponential decay of time-depend power and analysed with SEM. Implications on performance are analysed and discussed.

## Introduction

Cold plasma technology has been recently researched and its potentials validated, through functional prototypes, for a wide set of industrial applications including innovative refrigeration systems^1–3^, bacterial inactivation processes^4–7^, surface treatment procedures and many others^8–10^. There are several configurations^7,11,12^ that have been studied for the creation of related devices, capable of applying cold plasma to different industrial fields, from robotics to medical devices, from drones to space appliances. However, most available demonstrators are still in a prototype stage and little attention has been paid to long-term reliability, which is fundamental for increased safety, better operational stability, extended useful life and overall industrial and commercial success.

Continued use of corona discharge cooling devices in atmospheric air causes destructive and irreversible effects on the corona and ground electrodes. The environment where the discharge originates is considered an aggressive environment, as it is a highly oxidative medium. In the case of positive polarity, this is due to the presence of electrons that are directed towards the high voltage electrode, causing small shocks on its surface. On the other hand, the collector electrode is bombarded by chemical species such as O_3_, NO_x_ and SO_x_, which are generated during electrical discharge^13–16^. All these collisions of particles on the surface cause the activation of corrosion phenomena dependent on the exposure time and functioning conditions. The variation of the experimental and environmental conditions causes changes in the global power and overall performance of the device. Conditions such as local humidity, ambient temperature, ionization medium, applied voltage or degradation of the electrodes, can affect device stability by producing continuous electric arcs, hence causing a non-uniform discharge^17–19^. Because of different aforementioned variables, thin insulating layers may be generated during the use of these devices, which typically ends up with a smaller active area upon both electrodes. Progressively, the electrodes begin to lose their mechanical and electrical properties and the corona discharge efficiency is reduced. Materials selection and adequate management of degradation and corrosion phenomena plays, hence, a vital role.

Numerous investigations have focused on studying the erosion instigated by electrodes subjected to electric discharges with a high current density, point heating due to the presence of electric arcs and high applied voltage^20–22^. Under these conditions, micro-explosions are originated, due to a local concentration of high energy and overheating of the metal (leading to ecton production^22,23^). The initiation of these small explosions of metallic particles and the massive emission of electrons (generation of ecton) are decisive in the formation of oxidizing compounds. However, studies dealing with the degradation of surfaces exposed to positive and negative corona discharge are not common. In some investigations focused on corona discharge, the initial generation of an ecton is observed in the initial stage of the discharge, regardless of the polarity. Needle erosion has been studied, using typically copper cathodes in negative corona discharge applying Trichel pulses in atmospheric air for various configurations^24,25^. Nanometric craters appearing at the cathode needle have been reported, which indicates degradation of the electrode by an explosion emission mechanism. The value of the current density—5 × 10^8^ A/cm^2^—and the integral of the specific current action—4 × 10^9^ A^2^·s/cm^4^—of the Trichel pulse allow for an explanation about the reason for the erosion of the cathode, which appears to be originated by micro-explosions. Other tungsten, platinum, copper and lead electrodes have been studied, in negative corona discharges, with point-to-plane configuration and using an electrode gap of 3.1 cm in pure N_2_ and H_2_^26^. With a current density of 1 A/cm^2^, Weissler has reported the formation of craters, due to the mechanism of ecton erosion, as a consequence of the appearance of accumulated metal on the sides of the rounded tip.

In the case of positive corona discharge, there is much less information available, and the complex (and probably interwoven) corrosion mechanisms of the anode are still under debate. Recently, some authors have focused their research on understanding the reliability of wires as anodes^27,28^. Islamov and Krishtafovich have investigated the lifetime of the wires of different materials as a corona electrode. Tungsten, Au-coated stainless steels, Ni–Cr alloys, and silver alloys have been explored. Nichrome and silver and their alloys appear to present a very high average erosion rate, which limits their use to approximately 50 to 100 h (h). The most promising results have been described for tungsten and gold wires, which achieve lifetime of even some months. Comparing both materials, tungsten has a lower average anodic erosion rate than gold wires, being 0.24 μg/C and 0.60 μg/C, respectively. Finally, the trendline of the erosion rate of the wolfram filament presents an exponential model (S_W_ [μg/C] = (0.20 ± 0.10) ·exp (0.011 ± 0.020·Q). Considering the information available in the literature, the beginning of an analysis is proposed, in order to determine the optimal electrode for future implementation on an industrial scale.

In this research, the main objective is to analyze the erosion generated during the ionization process and, consequently, improve the reliability of an industrial electrohydrodynamic (EHD) cooling system. The system corresponds to the “TRAID” arrangement, presented in previous studies by our team^3,29^. Present study focuses on systematically evaluating the use of diverse wires as corona electrodes for Direct Current (DC) positive corona discharge in atmospheric air and takes into consideration different diameters and materials. Tungsten, nichrome, and stainless steel are considered due to their mechanical and chemical properties and potential for reliable and profitable performance in EHD cooling systems. The time-dependent power loss, related to electrical resistance, is measured to determine the rate of erosion, to predict the rate of corrosion and as a variable linked to overall performance and useful device life.

Furthermore, to understand the degradation process and the underlying mechanisms, electrodes exposed to discharge at different times are visualized by scanning electron microscopy (SEM), which leads to discovering varied corrosion mechanisms that affect corona discharge systems. Materials and methods employed are detailed below, before presenting and discussing main results.

## Materials and methods

**Figure 1:**
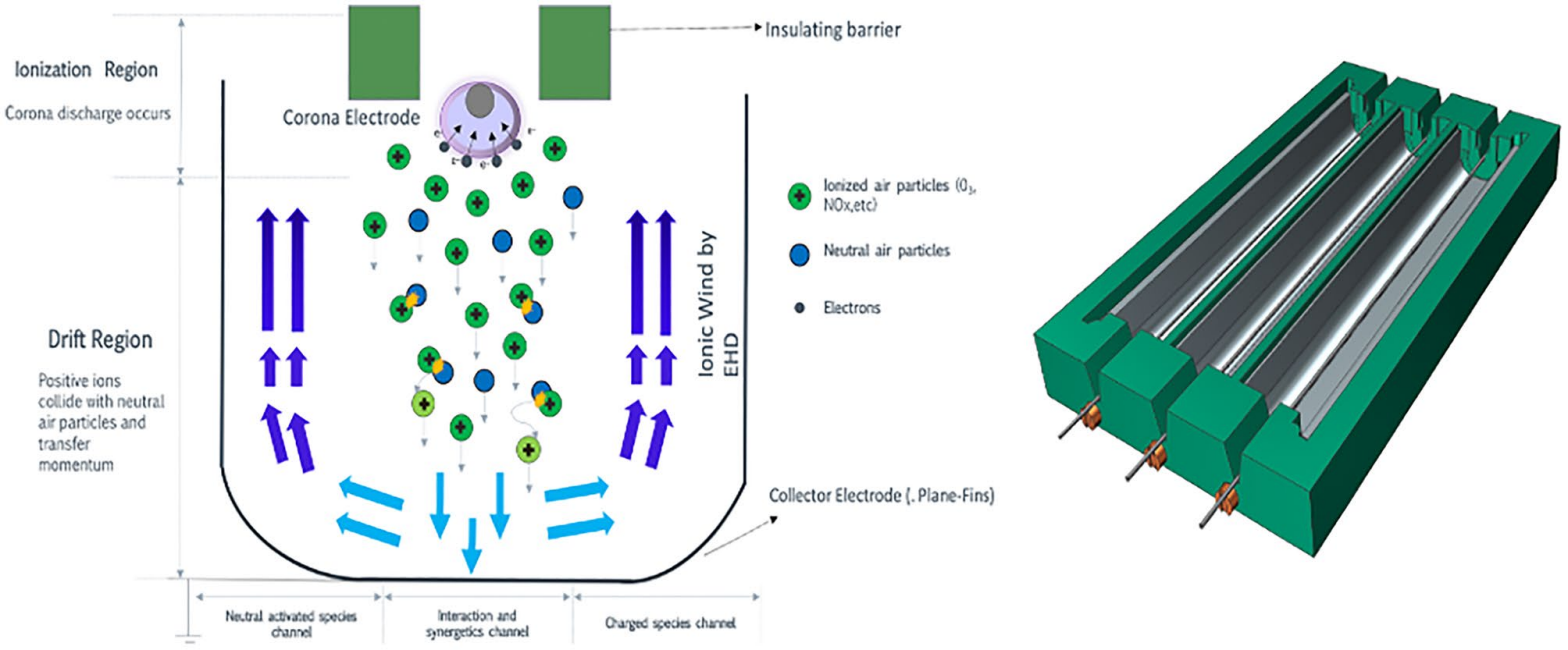
Schematic diagram of positive corona discharge and CAD design of TRAID configuration^3^.

This research has led to a comprehensive characterization of the TRAID configuration, which is a specific electrode system shown schematically in Fig. 1, performing as DC positive corona discharge system. The experimental electrohydrodynamic (EHD) pump follows a U-geometry and the corona electrode is made up of a wire fixed to the U-collector electrode at determined distance known as gap. First, the device is electrically characterized to record the loss of power over time. Subsequently, after corona discharge, a methodic study of filaments is performed by means of scanning electron microscopy (*Mitsubishi S-3400 N SEM system*). All tests have been carried out in a controlled system based on an environmental chamber (ICH110 Memmert) where temperatures of 20–25 °C have been set, a relative humidity of 30–40% and the pressure is considered 1 atm. Humidity parameter varies because experiments are executed with extraction to avoid high concentrations of generated species such as O_3_ and NO_x_. Otherwise, they will influence on the degradation of the electrodes adding new variables to our study.

### Experimental set-up

The configuration studied in Fig. 1 was tested. For designing the devices’ geometries, Siemens NX version 8.5 (Siemens AG, Werner von Siemens strasse 1, 80333, Munich, Germany) was employed, which is commercial computer-aided design and engineering software by Siemens PLM Solutions. The manufacturing of the prototype is carried out using a fused deposition modeling 3D printer, with commercial denomination Sigma R19 BCN3D. The device structure is made of polylactic acid (PLA) 850 filament and is used to hold both electrodes at a fixed distance. The device studied consists of two pieces which are assembled: the corona support and the metallic bulk ground plate with fins.

The different corona wire electrode materials include tungsten (W 99.95%), stainless steel (S30200) and nichrome (Ni_0.8_Cr_0.2_). These materials have been selected because they are electrical conductors and have good mechanical and chemical properties shown in Table 1. The wires measure 40 mm in length and diameters of 25, 50 and 100 µm are analyzed, at an electrode gap of 2 mm from the U-collector aluminium (Al alloy-7075) electrode. The U-collector electrode dimensions consist of an internal channel with a width of 8 mm and counts fins with a height of 6 mm. The corona wire electrode is set at the same distance from both fins.

**Table 1:** Mechanical and electrical properties of materials used as corona electrode.

Material	Density (g cm^−3^)	Electrical resistivity (µmΩ cm)	Thermal conductivity (W·m^-1^·K^-1^)	Melting temperature (°C)	Tensile strength (MPa)	Galvanic potential (V)
W	19.3	5.4	173	3410	550–620	− 0.39 to − 0.31
Ni_0.2_Cr_0.8_	8.40	108	13.40	1400	650–1100	− 0.17 to − 0.09
UNS 30200	19.3	70–72	16.30	1400–1420	510–1100	− 0.09 to − 0.01

### Performance evaluation

To study the wear or degradation of the different electrode materials and to obtain the erosion rates for each case, different device structures with the same geometry and parameters are needed and tested. Corona experiments are performed to study the response, performance, and stability of different materials during the positive electric discharge phenomenon. The current–voltage characteristic curves (CVCs) are studied for different times of exposure at a constant voltage. Consequently, the power and electrical wire resistance respect to time are obtained and studied. In this way, the impact of electrode erosion on the behaviour of the EHD system can be verified. The corona equipment used in this research includes a high voltage power supply Heinzinger LNC 10,000-5, where both electrodes are connected for positive DC discharge up to 6 kV, which produces a potential difference between the corona wire electrode and the collector electrode. The wire power is monitored using a data logger RS PRO TES-1384 and a digital multimeter FLUKE 115 TRUE RMS. Due to the micrometric sizes of the high voltage electrode, determining the components of the oxidized film and its thickness using chemical analysis and visualization techniques is a challenge. The studies of the surface for different materials, used as electrodes, are performed both before and after the corona treatment through SEM microanalyses.

## Results and discussion

### Power–time measurements

Tests are performed to determine the behaviour of different corona electrodes materials exposed, during relevant amounts of time, to positive corona discharge at high voltages typical from real operation. Under the corona discharge effect, the thermophysical properties of electrode materials play an essential role in erosion. Tungsten (W), nichrome (NiCr) and stainless steel (SS) are evaluated as potentially interesting materials to develop the experiments, because they are electrically conductive, have good corrosion resistance and are commercially available. In the experiments presented, the wires used as corona electrodes have the 40 cm in length and the diameters vary between 25, 50 or 100 µm. Current–voltage characteristics curves are determined for different times of exposure at a fix voltage. This voltage typically depends on the geometric parameters of the device: distance between electrodes, wire diameter and configuration. As time increases, the current decreases; as a result, higher values for operating voltages are required, which reduces the performance and life span of the devices.

Based on the different values obtained by the CVCs curves the power consumption for the different materials is determined. As the exposure time to the corona discharge effect is increased, the corona power decreases. The experiments stop when the break point of the wire is reached or when the number of sparks forms a continuous stream that creates a short circuit. In the case of 25 μm wires, the applied voltage is 4.7 kV and a current of 0.2–0.25 mA. Meanwhile, the 50- and 100-micron wires are subjected to 5 and 5.4 kV of voltage, respectively, and 0.2–0.25 mA of corona current. All filaments are tested from 1 to 1.5 W of initial power.

**Figure 2:**
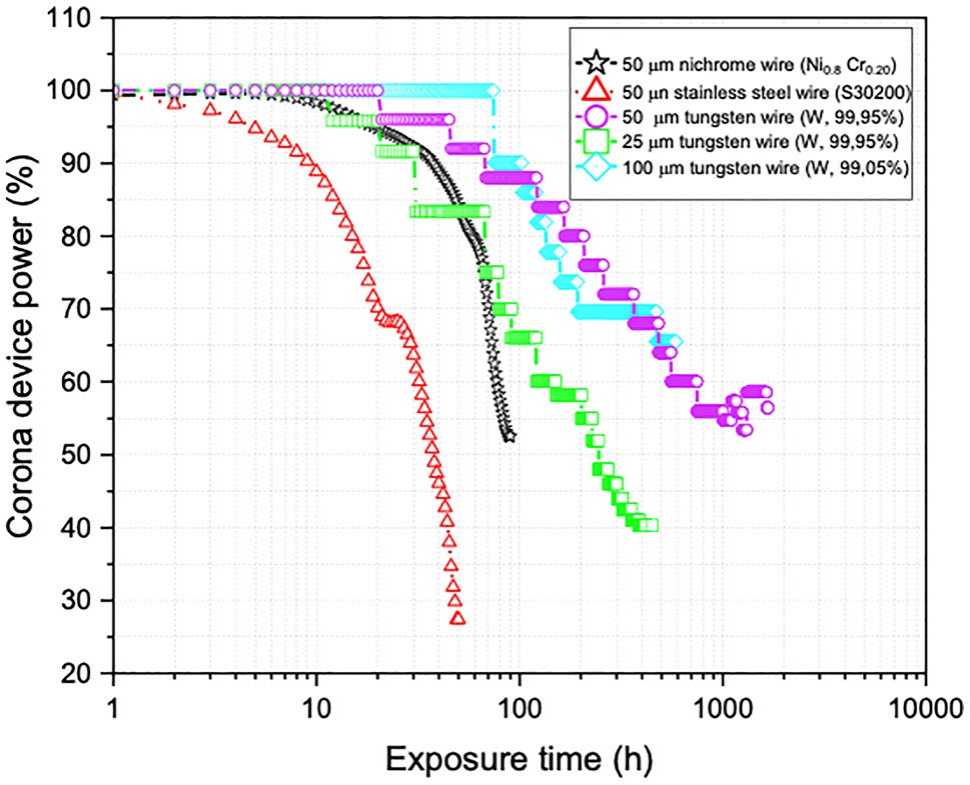
Power loss of different electrode wires under positive high voltage corona discharge.

To evaluate the different materials used as corona electrodes, a first round of experiments is performed using a 50-micron wire diameter for all of them. All curves represented in Fig. 2 are an average of the tests performed for each wire, in order to analyse power loss with increasing exposure of the devices to the corona discharge. For the 50 µm diameter wires both experiments of stainless steel and nichrome are stopped, as they break at 50 and 95 h, respectively. Tungsten wire does not break but the streamers formed make impossible the study when the 200 h are reached. We can observe how Ni–Cr and W wires have similar behaviour for the first 70 h which they only arrive at a power loss of 20%. After that point we can observe a drop of power for nichrome wire in its last 30 h of lifetime.

Stainless steel is considered to have a bad response to corona power consumption, as it breaks soon with a high-power drop. The misconduct of the nichrome and stainless-steel wires during the exposure of the discharge can be related to the boiling point of the alloys that constitute the material, which have low boiling temperatures with respect to the temperature at which the plasma is generated (< 10,000 K). Comparing all different materials for the same diameter, tungsten wire exhibits a better performance, as the wire does not break during any of the performed experiments and because it leads to the lowest values for the corona power reduction. Tungsten has a ceramic character that allows working at high temperatures, good resistance to aggressive environments and good mechanical performance. Therefore, tungsten is a proficient material to be used as an electrode. Subsequently, a second round of experiments is performed with tungsten wires and with varying diameters of 25 and 100 microns, in order to determine the useful life of these electrodes for its industrialization. As shown in Fig. 2, the tungsten with different diameter sizes presents a similar power drop around 60 h of use for all studies. Regarding the 25-micron wire diameter, when its power drops by up to 40%, it stabilizes for up to 450 h, when continuous arcs or streamers begin to form. On the other hand, the 50- and 100-micron corona electrodes stabilize after an exposure of about 200 h. In the case of the 100-micron corona electrode, the tests are stopped from 600 h of exposure of the discharge due to the generation of continuous electric arcs. Meanwhile, the 50-micron wire can overtake up to service times exceeding 1000 h. In the last two cases, the tests are stopped with different exposure times of the discharge to visualize these filaments by scanning electron microscopy.

### Degradation of W, stainless steel 302 and nichrome corona wires electrodes: erosion rate (E)

Anode degradation is investigated in positive corona discharge in atmospheric air. The micro-craters produced by the ecton generation are related to the mechanism of erosion and oxidation in corona filaments. Investigation of anode degradation rate is developed for several different configurations shown in Table 2. Lifespan testing initiates with new W, Ni–Cr, and stainless steel (S30200) filaments of 50 µm. Nichrome and stainless-steel wires show an increase in electrical resistance in a few hours of discharge exposure, causing breakage or streamer formation. The generation of a uniform oxide layer is macroscopically visible in both previous materials with an ash gray color in the initial 40 h of testing. Regarding tungsten wire, in the first 80 h there is no appreciable change for its operation. However, after this period, the resistance of the filament begins to increase slowly until stabilizing to 200 h of procedure, when the gray-whitish generated oxidation layer is already visible. This behavior of the tungsten filament is verified for other sizes of 25 and 100 µm, respectively. Following the similar trend, an alteration in electrical resistance occurs within the first few hours but stabilizes over time until the electrical discharge is not uniform. For the TRAID configuration, it can be determined that the service life of tungsten filaments exceeds 1000 h but with significant damage due to the corrosion mechanisms that these wires develop.

**Table 2:** Experimental considerations, materials, and average degradation rate of W, Ni_0,8_Cr_0,2_ and stainless steel.

Material	D (µm)	Number assays	$\Delta$ V (kV)	Exposure time, t_f_ (h)	Corona power reduction (%)	Final state	Final current, I (µA/cm)	Q_f_ (C/cm)	e_a_ (µg/C)
Stainless steel 302	50	12	4.75	50	72.7	Rupture	15	4	29
Ni_0.8_Cr_0.2_	50	8	4.75	95	48	Rupture	32.5	11.1	7.6
Tungsten	50	18	4.5–4.75	1630	56.5	Stopped	50	300.42	0.92
	25	8	4–4.3	450	40.3	Stopped	27.5	44.5	3.7
	100	10	4.9–5.2	600	65.2	Stopped	40	86.4	5.3

The erosion rate is a difficult parameter to estimate because numerous phenomena overlap during corona discharge. Several authors such as Islamov and Krishtafovich^27^ estimated the erosion rate of various materials in their configuration. They related to this physicochemical phenomenon with the volume of filament subjected to discharge, the intensity, and the time of exposure. The average erosion rate formula *e*_*a*_ for a wire is expressed in μg/C and is shown below:

1 $${e}_{a}= \frac{1}{Q}{\int }_{0}^{Q}E dQ= \frac{\Delta {m}_{a}}{Q}=\frac{{A}_{d}}{Q}\cdot \left(1-\frac{{R}_{0}}{{R}_{a}}\right)$$

where E is the global rate of erosion as a function of Q(t) which is the charge transferred per unit length of the cable in C/cm. The charge transferred per unit length is defined as $\mathrm{Q}\left(\mathrm{t}\right)={\int }_{0}^{\mathrm{t}}\mathrm{I dt}$, where I is the current of the corona filament per unit length in µA/cm. On the other hand, A_d_ is the association with the cross section and the density of the electrode material, A_d_ [µg/cm] = 10^6^ · $\uppi \cdot$ (r [cm])^2^ · $\uprho$ [g/cm^3^]; R_0_ and R_a_ are, respectively, the initial and final values of the resistance per unit length of the electrode in the active exposure zone of the corona discharge. Estimating the erosion caused during the corona discharge process is a complicated method of determining when it depends on time. By improving the accuracy of this degradation rate, successive measurements can be used. It is considered that the overall erosion rate is proportional to the derivative of the corona electrode resistence R_0_/R in relation to the minority transferred charge Q.

2 $${E}_{i+\frac{1}{2}}=\frac{{A}_{d}}{{Q}_{i+\frac{1}{2}}}\cdot \left(\frac{{R}_{0}}{{R}_{i}}- \frac{{R}_{0}}{{R}_{i+1}}\right)$$

**Figure 3:**
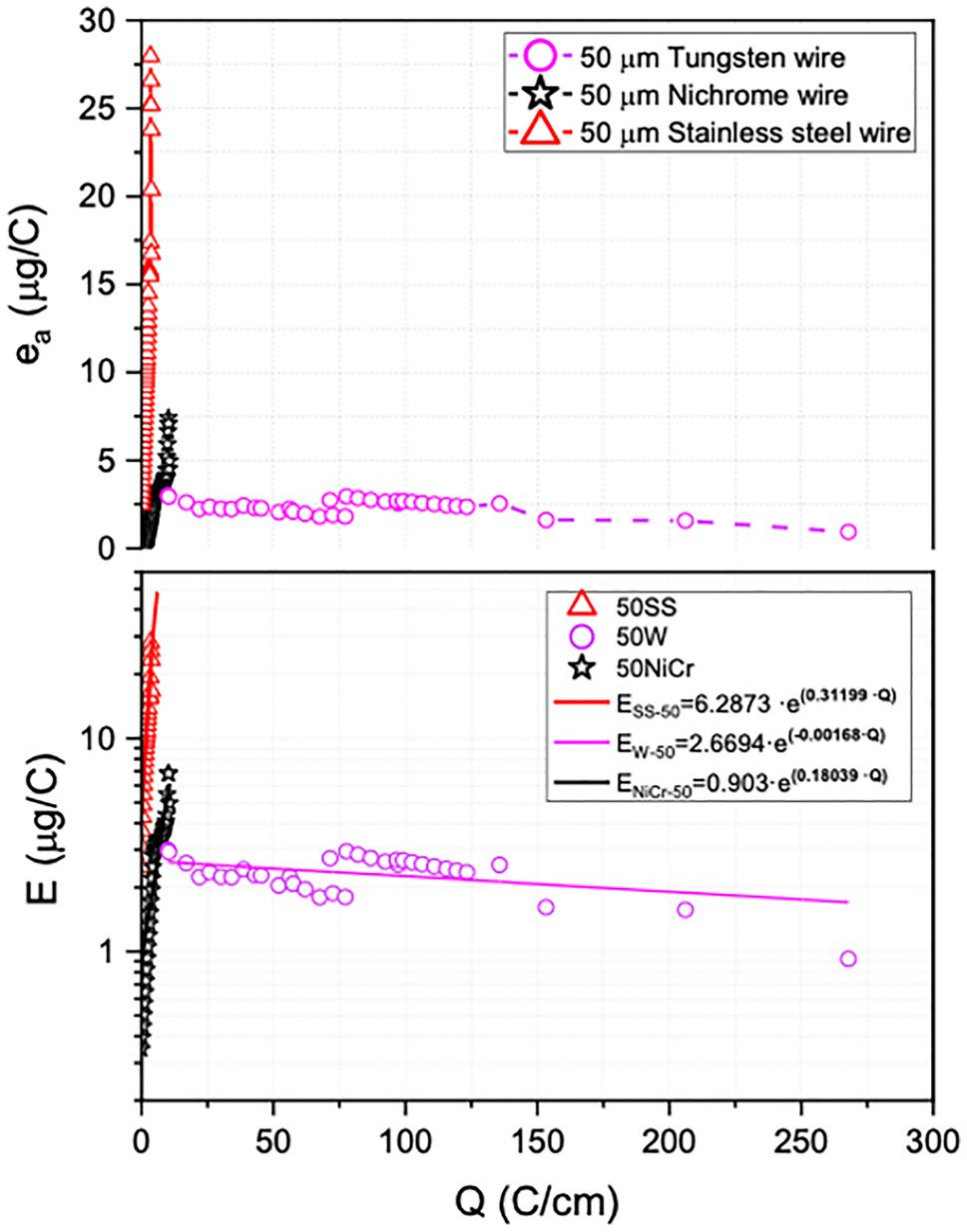
Analysis of tungsten, nichrome and stainless steel as corona electrodes relating average erosion rate (e_a_) and erosion rate (E) depending on transferred charge. All corona electrode assays are shown in Table 2.

As previously discussed, the erosion phenomenon of the corona anode is one of the main reasons why filament rupture occurs. In Fig. 3, the worst degradation behavior of stainless steel is determined, presenting a rupture at Q = 3 C/cm and an average erosion rate of 29 μg/C. A similar behavior is shown by the nichrome electrode which has a limited useful life, breaking at a transferred charge of 16 C/cm. However, the tungsten electrode has an average erosion rate 1.9 μg/C that remains unaffected with the exposure time, being the lowest of all electrodes.

The erosion rate is approximated exponentially for all the cables studied. An exponential regression and interpolation model of all the data obtained by the data logger is created. The erosion rate for 50 µm tungsten, nichrome and stainless-steel filaments is determined considering the model fit errors and a coefficient of determination R^2^ that is in a range from 0.61 to 0.88.

$$ \begin{aligned} {\text{E}}_{{{\text{SS}} - {5}0}} \left[ {{\mu g}/{\text{C}}} \right] \, & = ({6}.{2873 } \pm { 2}.0{6}0{57}) \, \cdot{\text{e}}^{{(0.{31199 } \pm \, 0.0{97}0{2}\cdot{\text{Q}})}} \\ {\text{E}}_{{{\text{NiCr}} - {5}0}} \left[ {{\mu g}/{\text{C}}} \right] \, & = (0.{9}0{134 } \pm \, 0.{41284}) \, \cdot{\text{e}}^{{(0.{18}0{39 } \pm \, 0.0{4647}\cdot{\text{Q}})}} \\ {\text{E}}_{{{\text{W}} - {5}0}} [{\mu g}/{\text{C}}] \, & = ({2}.{6694 } \pm \, 0.0{928}) \, \cdot{\text{e}}^{{( - 0.00{168 } \pm \, 0.00{15}\cdot{\text{Q}})}} \\ \end{aligned} $$

**Figure 4:**
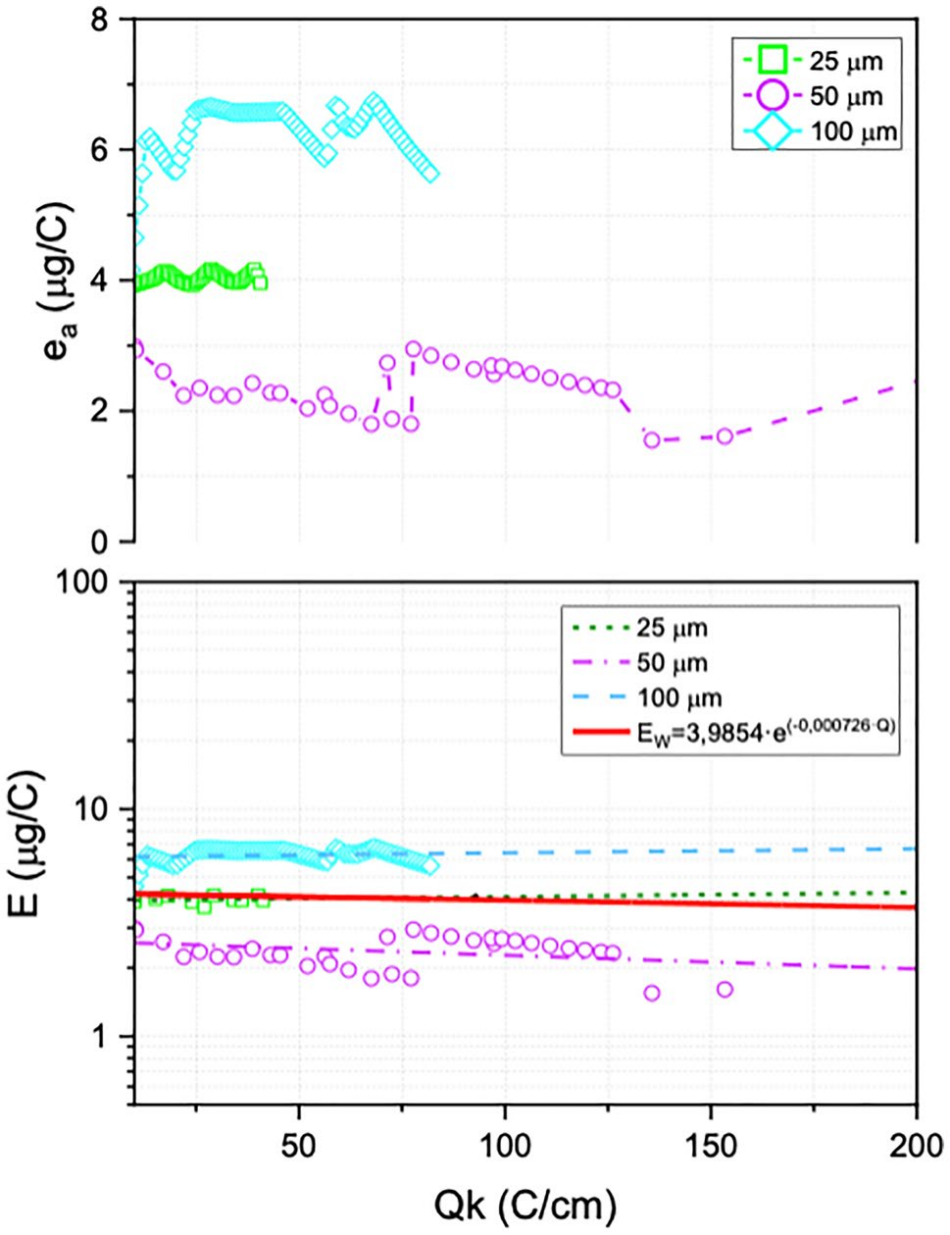
Average erosion rate (e_a_) and global erosion rate (E) depends on charge transferred per unit length Q considering different tungsten wire diameters.

The results of the average erosion rate for the different W wires studied dependent on the charge transfer are shown in Fig. 4. It is observed that an average erosion rate for the W filament of 25 µm remains approximately 3.7 µg/C. On the other hand, the 50- and 100-µm anodes, unpredictably, do not have a similar average erosion rate in most of the tests performed. The 50-micron wire is remarkable because it has a low erosion rate compared to the other two wire sizes, being from 1.9 to 0.9 µg/C. Instead, the erosion rate performance for the three different diameters of tungsten shows a stable and constant trend when a charge transfer of about ~ 25 C/cm is exceeded. However, the erosion behavior of tungsten shows an appreciable difference with our electrode arrangement being higher than that reflected in the literature. These results may be associated with the prototype geometry and the different applied electrical parameters. For the TRAID configuration it is estimated that it has an erosion rate exposed below:

$$ {\text{E}}_{{\text{W}}} [{\mu g}/{\text{C}}] \, = ({3}.{9854} \pm \, 0.{1628}) \, \cdot{\text{e}}^{{( - 0.000{726 } \pm \, 0.0000{52}\cdot{\text{Q}})}} $$

In general, it is determined that nichrome and stainless steel are not competitive electrodes for use in non-thermal plasma in atmospheric air (for corona discharge applications) due to their accelerated corrosion and their limited-service life. On the other hand, tungsten is still the best material due to its refractory behavior and good resistance to degradation. However, due to the aggressive environment where the discharge has effect, the erosion and oxidation mechanisms are the main dominant phenomena that produce an electrode lifespan of 1630 h without cleaning and maintenance.

### Accelerated degradation under corona discharge phenomena

While studying the degradation of electrodes under corona discharge, it has been identified time as a critical factor. The amount of time needed to obtain consistent values for service life in electrodes could require years of tests. Due to this, the importance of accelerating the amount of damage generated on electrodes while being tested and being able to quantify that damage has been one of our goals.

Fourteen 50-micron filaments have been subjected to accelerated degradation tests using a voltage applied of 5.3 kV and a current between 0.35 and 0.4 mA. The acceleration of time damage on electrodes has been done by calculating the cumulative energy density that the electrode handles in MJ/cm^2^. By using that parameter, we can on one side, obtain typical values of cumulative energy density in which the electrode breaks and on the other side set a strategy to increase the energy density the electrode handles in each time step to reduce testing length.

The selection of energy density has been made since this energy has been proved to be the driving force of all the chemical reactions and therefore it directly influences service life^30^. Energy density has been chosen instead of energy because it’s easier to scale it to different kind of electrodes by taking its surface as reference. Also, the exposed surface is the place where most of the reactions take place in between chemical elements, molecules, electrodes, etc.

The steps to calculate the energy density are shown in Eqs. (4), (5) and (6).

4 $$W=I\cdot V$$

5 $${\rho }_{W}=\frac{W}{{S}_{c}}$$

6 $${\rho }_{E}=\frac{1}{{10}^{6}}{\int }_{0}^{t}{\rho }_{W}\cdot dt$$

where W refers to power in [W], I refers to current in [mA], V refers to Voltage in kV, S_c_ refers to the electrode surface in cm^2^, ρ_W_ refers to power density in W/cm^2^ and ρ_E_ refers to energy density in MJ/cm^2^.

**Figure 5:**
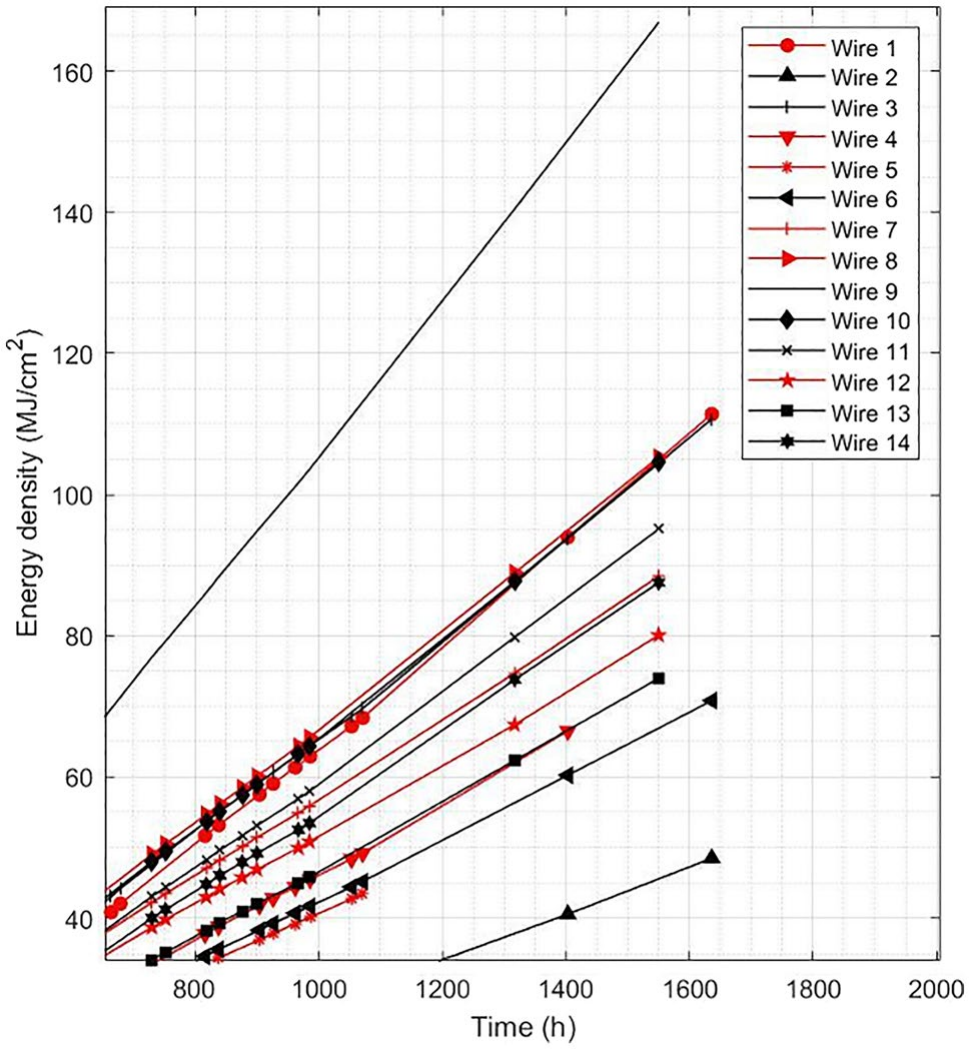
Cumulated energy density (MJ/cm^2^) recorded for 14 wire electrodes tested for 1550–1630 h.

Integrating Eq. (6), for the experiments done on the degradation benches, the results shown in Fig. 5 are obtained.

These test benches have been studied at a higher service power to obtain the limit of accumulated energy density that an electrode can handle. At approximately 1550 h, the maximum value obtained is 166 MJ/cm^2^.

**Figure 6:**
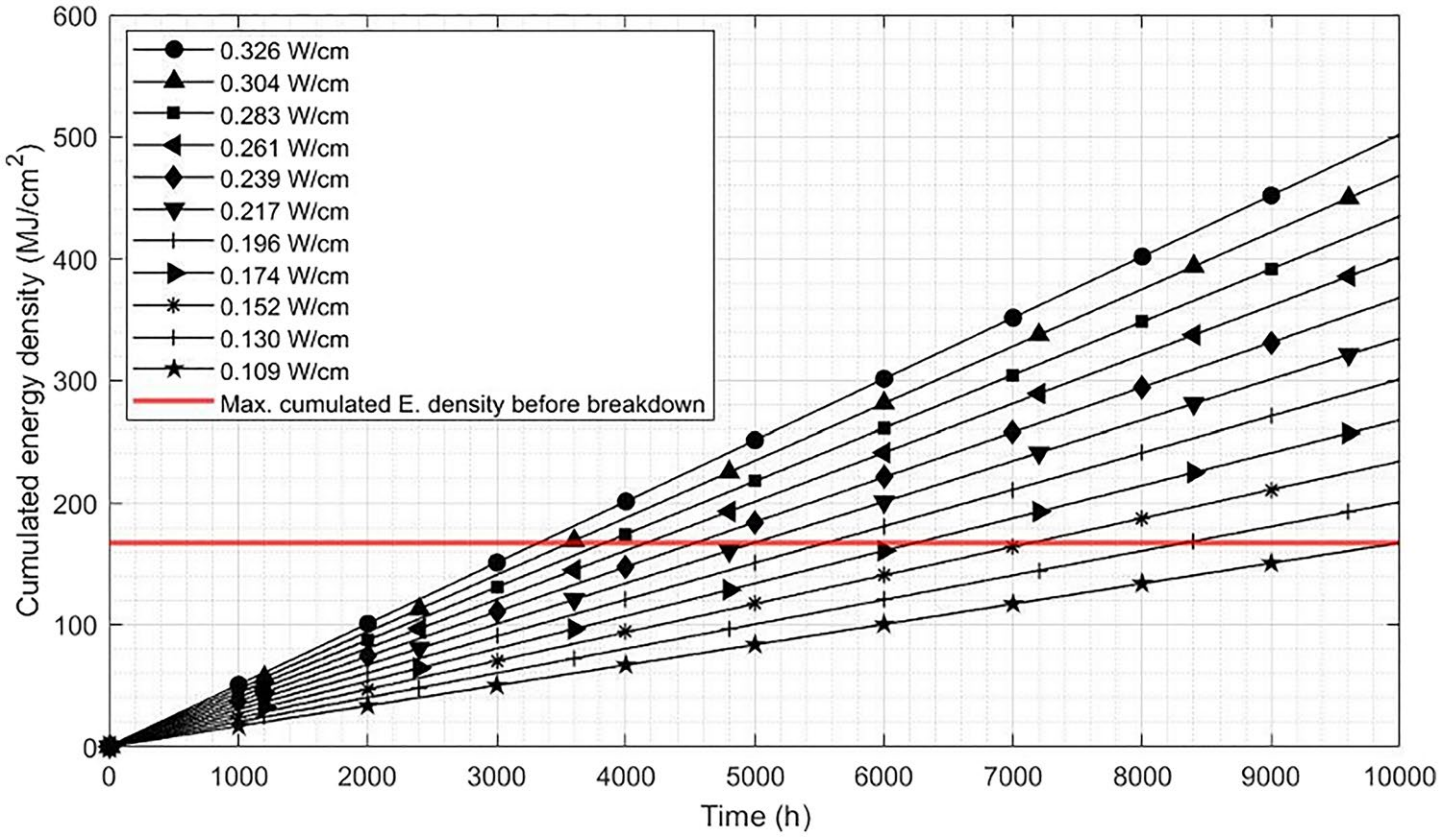
Service life prediction for different levels of power density on wire electrodes.

With that cumulative energy density result and the power loss on electrodes we can give a rough service life estimation at designed operating condition for electrodes^31^. This result has been calculated for several power densities that can be seen in Fig. 6 (in this case, we use linear power density in W/cm for design purposes).

The horizontal red line represents the current cumulative energy density limit which grows while the time the test electrodes are exposed to high power grows. Right now, the estimated service life for electrodes at average power is 4980 h. At minimum power it can be increased up to 10,000 h and at maximum power it decreases to 3300 h.

To improve the reliability of this model and for being able to obtain a higher cumulative energy density limit in a shorter period, it is planned to accelerate even more the test by increasing the energy that the electrode handles. Several ways to increase that energy have been studied^32^. The easiest one is increasing the voltage in between electrodes but it can make the discharge less stable. That is why in the future tests will be done in other atmosphere with higher temperature and lower pressure to increase the energy density while keeping the discharge stable.

The aim of increasing the energy density is to reach an acceleration factor of more than 10 (predicted hours/tested hour), being this number right now is around 3.2.

### Surface morphology: SEM analysis before and after exposure corona discharge

**Figure 7:**
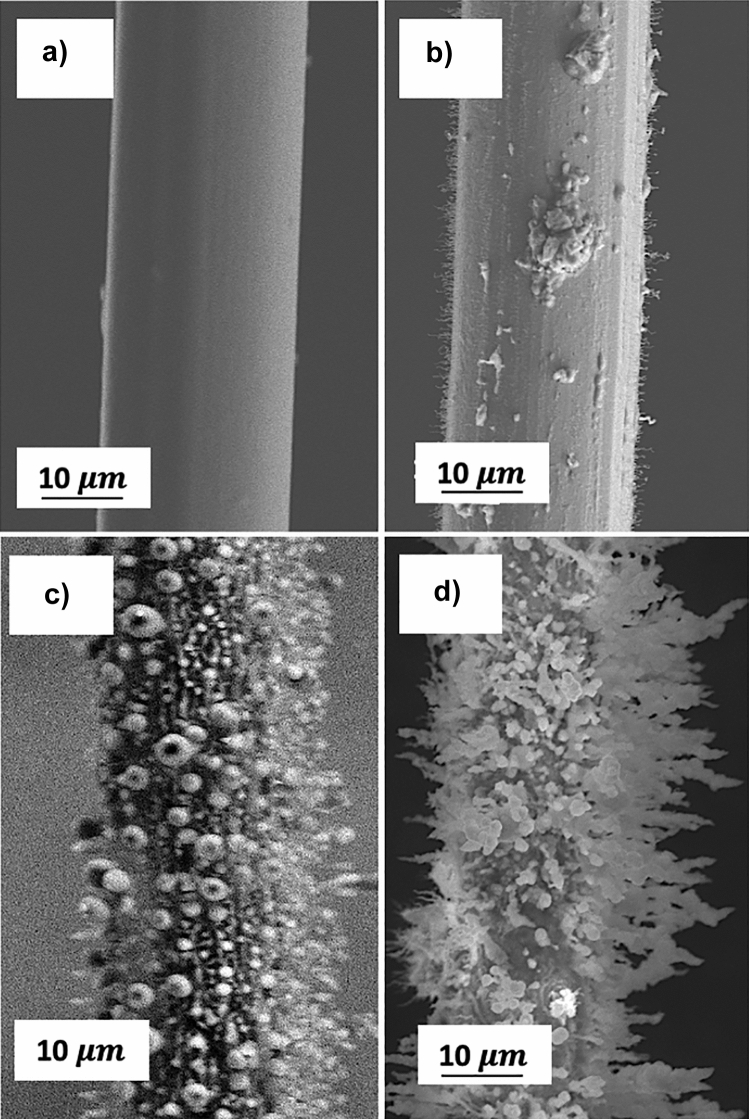
Tungsten wire of 25 μm exposed at (**a**) 0 h, (**b**) 20 h, (**c**) 280 h and (**d**) 450 h by atmospheric corona discharge.

Corona electrodes are analyzed using scanning electron microscope (SEM) images. The study of different diameters of tungsten wires are observed over the central length exposed to the corona discharge. First, the diameter tungsten wire of 25 µm for different hours is visualized obtaining images like the ones shown in Fig. 7. The advance of oxidation is observed, according to the formation of plasma over time in a new filament (see Fig. 7a), beginning to originate oxides on the surface not uniformly after 20 h (Fig. 7b).

Considering a longer exposure time, reaching up to 400 h of use, an irregular oxidative film based on spherical and fractal structures can be seen, mostly in the ionization zone, as shown in Fig. 7c and d. This corrosive phenomenon causes significant changes in the discharge as well as in the direction and intensity of the electric field due to the presence of this film, causing a smaller effective discharge area. Therefore, electric arcs and streamed are created and are tried to go through the corroded film. It is important to consider that although this continuous formation of oxides due to erosion and the oxidative medium where it occurs is quite unstable and most of the oxides volatilize.

**Figure 8:**
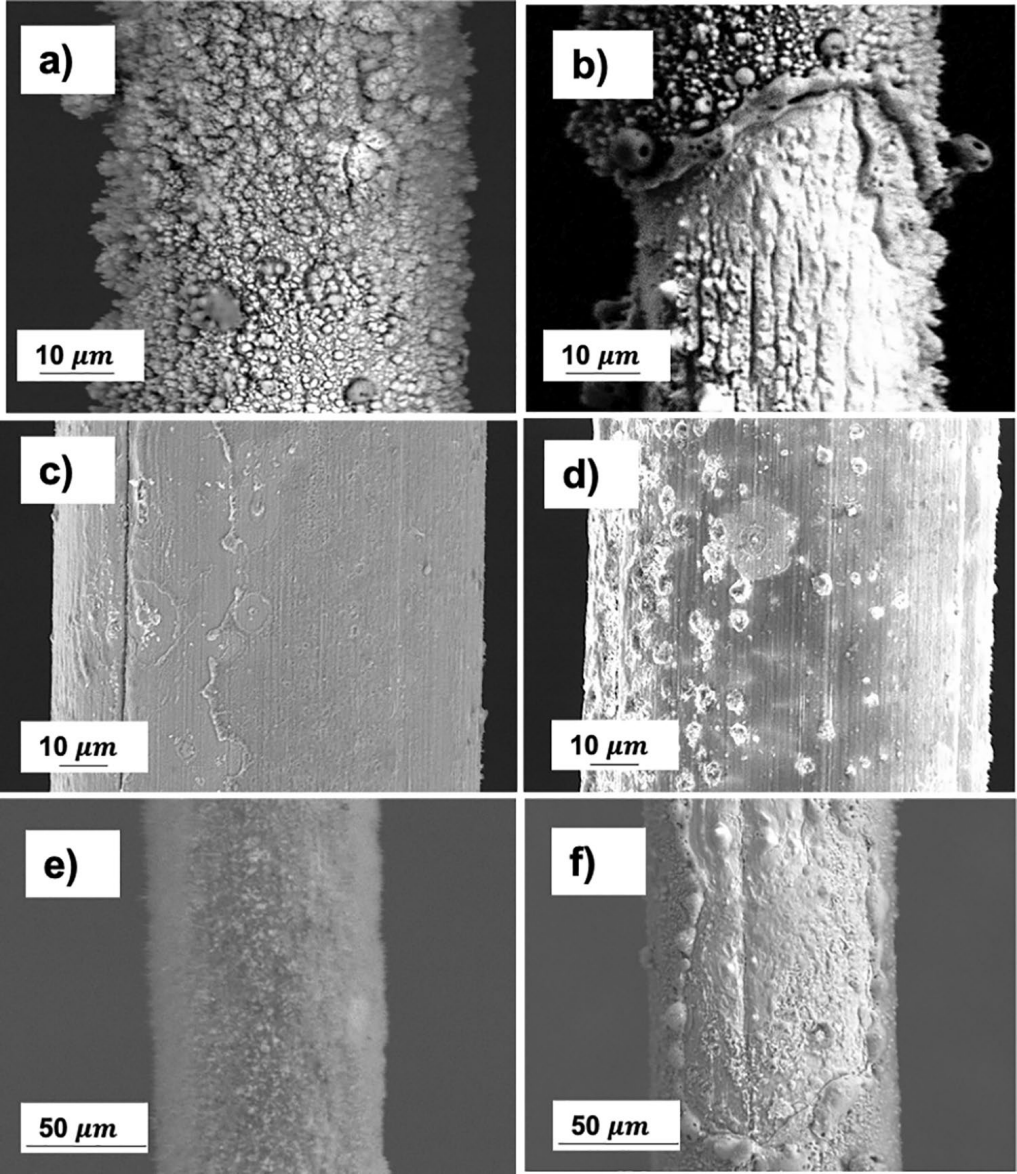
Tungsten filament of 50 μm after treatment at (**a**, **b**) 250 h which a dense and non-uniform oxidation layer can be observed on the entire external surface, observing in some areas the rupture of the oxide layer due to the instability of the discharge. In the micrographs (**c**, **d**), the 100 microns tungsten anode is visualized at different exposure hours, 40, 180 h respectively, where bombardments begin to occur by the electrons. (**e**, **f**) SEM images showing W filament of 100 μm undergoes a corona discharge at 470 h, showing stable layer of oxides with a pattern similar to velvet and fused areas due to the generation of punctual micro-arcs.

The tungsten anode of 50 and 100 µm after corona discharge is shown in Fig. 8. An oxide layer with a rough surface form on the 50 µm filament after 250 h of discharge over the entire surface of the anode (Fig. 8a,b). Two significant regions are shown: on the one hand, areas with stable oxide layers around the anode; on the other hand, sections affected by the impact of an arc which has caused the rust shell to break and local fusion in that area.

On the exposed surface in front of the ground electrode, nanometric craters are visualized at 40 and 185 h (see Fig. 8c,d). Around these micro-explosions, nanometric oxides begin to form, overlapping several effects: local temperature changes due to the impact of electrons with temperatures above 10,000 K, atmospheric air as a corona discharge medium that induces accelerated oxidation and the application of a high electric field that directly influences the degradation mechanism. An exposure time of 470 h is evaluated in the SEM images (Fig. 8e,f) in which two significant regions are also observed where the oxide presents a uniform velvet pattern and fused areas that cause discontinuities in the discharge.

**Figure 9:**
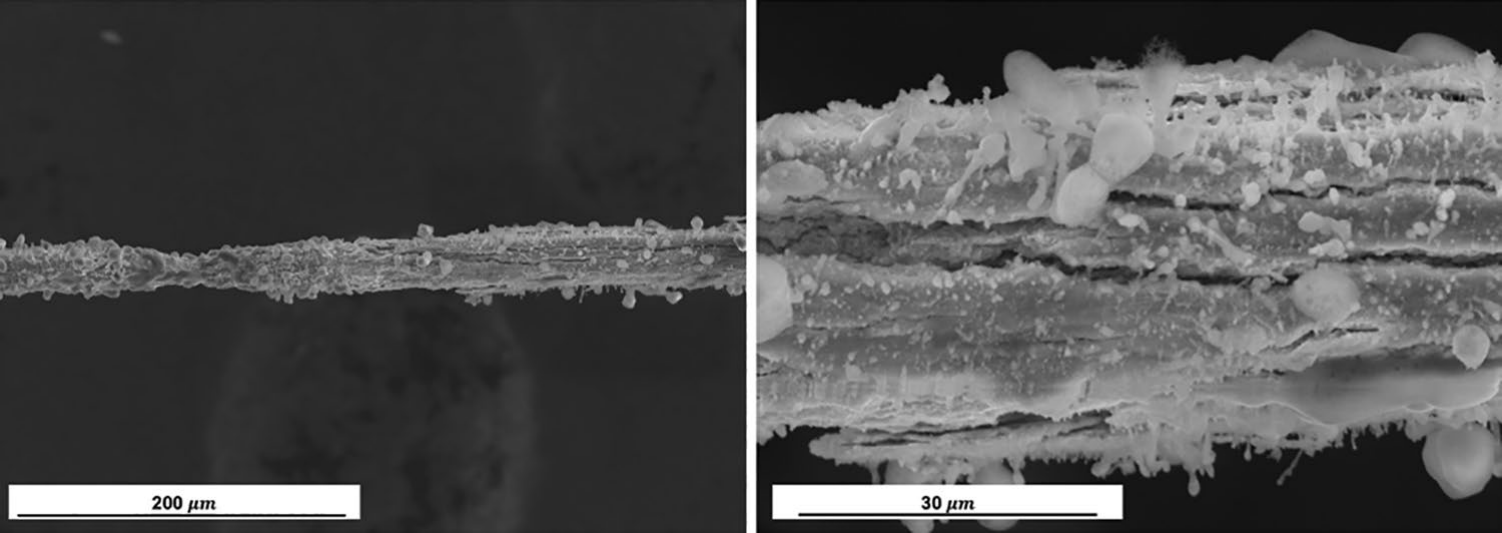
Thickness change of W wire of 25 µm after 300 h in service due to erosion and corrosion triggered in the process. There is a reduction in section that may be due to the combination of localized detachment by micro-arcs. Also due to plastic deformation favored by focused temperature increases in specific arc-areas. Necking has been observed in some filaments without appreciable degradation.

In addition to the oxidation film created, electron bombardment-induced erosion results in significant mass loss over time. It can be clearly seen how the wire in a certain area experiences a considerable decrease in the cross-sectional area (Fig. 9). This could be because of combination of the localized losses of oxide layers, due to microarcs produced, and plastic deformation promoted by the operating temperature during these phenomena. The Ductile–Brittle Transition Temperature (DBTT) of tungsten is in the interval of 200–450 °C^33,34^, but it depends on metallurgical factors that can do it to change. Plastic deformation in tungsten only is possible if the temperature is over this DBTT. Therefore, the reached temperature in these areas has been higher than the transition temperature. This change in dimensions directly influences the operation of the discharge, this area being a critical point of electrode breakage. The presence of longitudinal cracks can be caused by the wire manufacturing process and the excessive tension applied to fix the filament on the prototype. These structural imperfections also influence corrosion, accelerating the entire process.

## Conclusions and perspectives

The industrial success of atmospheric non-thermal plasma devices relies on adequately taking the deterioration of active electrodes into account. In this study, the degradation of the surfaces of corona electrodes has been demonstrated for most materials used as active electrodes in ionic wind generation devices. For most materials, this degradation starts after a short period of use, which importantly limits electrode life. A superposition of corrosive effects such as the impact of electrons on the surface at high temperatures and atmospheric air as a means of ionization cause accelerated corrosion in the vicinity of the wire. This corrosion leads to misshapen and non-uniform structures along the plasma exposed wire. Numerous materials are studied as active electrodes. Nichrome, stainless steel, and tungsten of 50 µm are tested with the same configuration and power to estimate their useful lifetime in non-thermal plasma devices. The power loss of more than 50% in nichrome and stainless steel is verified after 50 h, causing the breakage of the wire in most of the tests carried out.

Therefore, tungsten is studied for different wire sizes (25, 50 and 100 µm) where its loss of properties after corona discharge is evaluated for several hours. The W filament can be used for approximately 1000 h with a loss of power less than 60% in the wires which are analysed. The aim is based on determining the total service life thought an energy model related to the power of the device is proposed. With the experimental tests carried out, they allow us to determine an approximation of 4980 h of operation for high powers. However, a low power density of the device allows us to ensure a lifespan of more than 10,000 h, being competitive with other industrial methods.

According to our experiments, tungsten is one of the corona electrodes with the greatest potential for industrialization according to our research and other authors who determine its possible use. It is important to highlight that the key to success of corona discharge electrode materials is, not only related to the capability of withstanding the discharge in service, but also to their processability and cost. Although tungsten provides the best compromise among the material studied, corona discharge systems are not yet competitive, in terms of lifespan, when compared to other conventional refrigeration systems.

However, corona discharge cooling devices present important advantages in terms of size, weight and ease of operation, which motivates further studies linked to achieving a deeper understanding of electrode degradation phenomena and to micro and nanomanufacturing post processes for improving the electrode endurance to corrosion. For all these reasons, and considering the aforementioned advantages of ionic wind devices, the most important challenge is to increase the service life of the electrodes.
